# Mpox in a patient with AIDS: clinical management with tecovirimat and surgical correction of unaesthetic scars^⋆^

**DOI:** 10.1016/j.abd.2024.11.001

**Published:** 2025-04-07

**Authors:** Kananda Kesye Sousa Nunes, Carlos Alberto Chirano Rodrigues, Beatriz Costa Cardoso, Iêda Lúcia Santos Magno, Mathias Gama de Aguiar Ferreira, Sinésio Talhari

**Affiliations:** Department of Dermatology, Fundação Hospitalar Alfredo da Matta, Manaus, AM, Brazil

*Dear Editor,*

A 33-year-old male patient, single, born and raised in Manaus, Amazonas, Brazil, sought medical care due to ulcerated lesions on the face, chest, upper limbs, gluteal region, and anorectal mucosa (Figure 1, Figure 2). He also reported recurrent fever and anal pain. He had been treated with several medications, such as anti-inflammatory drugs, topical, and systemic antibiotics, for over a month, without response. He had no neurological or respiratory symptoms and denied comorbidities.

**Figure 1:**
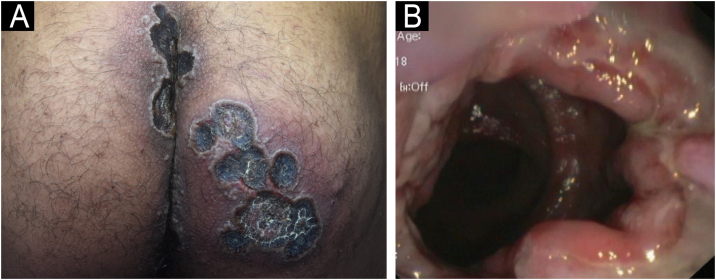
(A) Ulcero-necrotic lesions on the buttocks. (B) Colonoscopy image: ulcerative proctitis.

**Figure 2:**
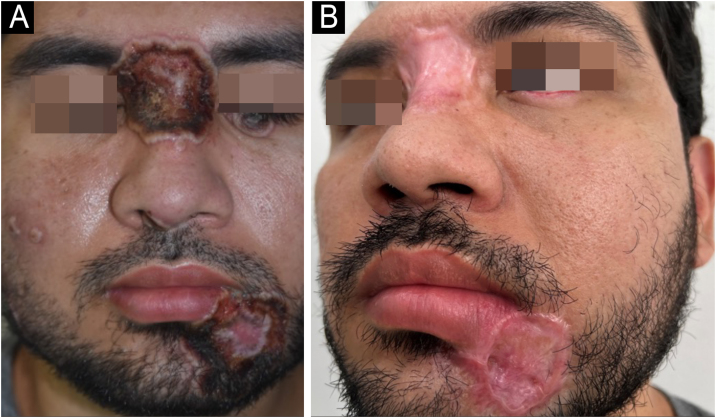
(A) Vesicopustular lesions in the malar region; ulcer in the lower eyelid, glabella and labiomental region. (B) Two months after treatment.

Laboratory tests were requested, and the main results were: qPCR for Monkeypox ‒ detectable virus; Anti-HIV ‒ reactive; VDRL ‒ 1:256; cerebrospinal fluid analysis ‒ no findings suggestive of neurosyphilis; colonoscopy – ulcerative proctitis (Fig. 1). The anatomopathological examination of the ulcerated skin lesion showed epidermal necrosis, ballooning of keratinocytes, Guarnieri inclusion bodies and a dermal inflammatory infiltrate consisting of histiocytes, lymphocytes and neutrophils (Fig. 3).

**Figure 3:**
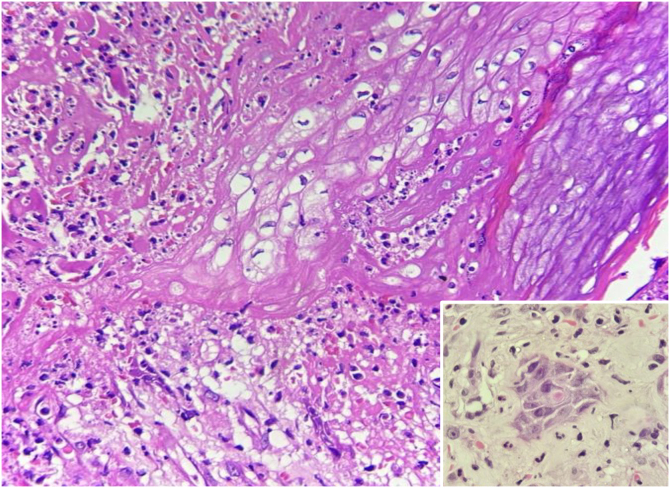
Histopathology: ballooning of keratinocytes (Hematoxylin & eosin, ×400). Inset: Guarnieri’s inclusion bodies.

The initial treatment included benzathine penicillin, a single dose of 2.4 million IU, with a subsequent adequate decrease in VDRL; and antiretrovirals (Dolutegravir, Lamivudine and Tenofovir), in addition to supportive measures.

Given the worsening of the skin lesions, anal pain, and extension of the anorectal involvement, the patient was hospitalized in isolation, submitted to colostomy and, seven days after admission, started treatment with tecovirimat, 600 mg every 12 hours, for 14 days.

Approximately 20 days after starting therapy with tecovirimat, associated with systemic antibiotic therapy for secondary infection, healing of the skin lesions was observed (Fig. 2), without adverse events.

The regression of the skin ulcers evolved with hypertrophic scars, mainly in the glabella and labiomental region. At this stage, surgical repair was indicated, performed ten months after hospital discharge.

Initially, excision and correction of the labiomental scar were performed with an advancement flap and the creation of a Burow’s triangle (Fig. 4). Subsequently, the glabella scar was corrected.

**Figure 4:**
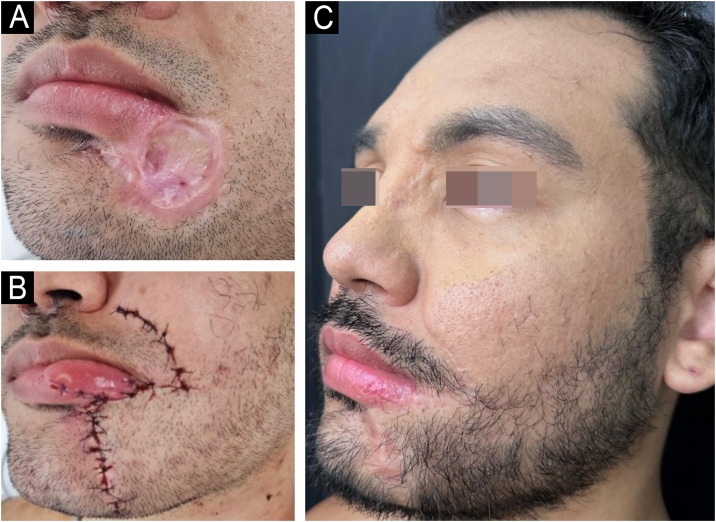
(A) Scar in the left labiomental region. (B) Immediate postoperative period. (C) Late postoperative period.

Mpox is caused by the Monkeypox virus – a double-stranded DNA virus belonging to the Orthopoxvirus genus, Poxviridae family. Clinically, vesicopustular, ulcerative-crusted, or ulcerative-vegetative lesions are observed; fever, myalgia, headache, and lymphadenopathy may be present.^1^, ^4^, ^5^, ^6^, ^7^, ^8^, ^9^

The disease has been endemic in African countries for decades, causing occasional outbreaks restricted to the continent. However, in 2022, and more recently in August 2024, international epidemic outbreaks were recorded, with the spread of strains 2b and 1b respectively, and the World Health Organization (WHO) declared the disease a “Public Health Emergency of International Concern” in both scenarios. Strain 1b, detected in the Democratic Republic of the Congo, has been shown to be more virulent and lethal, with a particularly severe evolution in immunocompromised individuals, children, and pregnant women. There are no records of the circulation of this strain in Brazil to date. Since 2022, more than 10,000 confirmed or probable cases of Mpox have been reported in Brazil, including 16 deaths in immunosuppressed individuals.^5^, ^10^

Before the outbreaks observed in 2022, it was assumed that transmission was mainly related to contact with skin lesions and the respiratory tract. However, the demonstration of the presence of the virus in semen and rectum indicated sexual transmission as a risk factor, mainly among men who have sex with men (MSM); 91% of the patients with Mpox are male and more than 50% are MSM. Mpox is also a risk factor for HIV – coinfection occurs in more than 40% of the cases.^2^, ^3^

The patient in this report was diagnosed with HIV, with CD4 T-lymphocytes at 44 cells/mm^3^ and a viral load of 94,000 copies/mL, at the time of the Mpox diagnosis. Sexual history revealed unprotected anal sex with other men.

Patients who are coinfected with Mpox and HIV, mainly with a low CD4 + T count (< 200 cells/mm^3^), have a longer disease duration. The lesions may be larger and have a necrotic appearance, as observed in this case. Bacterial infections, sepsis, and death may also occur. Some authors consider Mpox to be an opportunistic infection or an AIDS-defining disease.^3^

Mpox treatment consists of general care and supportive measures. Recently, on an emergency basis, the Centers for Disease Control and Prevention (CDC) authorized the use of tecovirimat, brincidofovir, cidofovir, and trifluridine (ophthalmic solution). In Brazil, in 2022, the National Health Surveillance Agency (Anvisa, *Agência Nacional de Vigilância Sanitária*) approved the compassionate use of tecovirimat only in severe cases of the disease, as in the case reported herein. This drug inhibits the VP37 protein, responsible for virion enveloping, preventing viral replication. It is presented in 200 mg capsules, for oral administration, at a dose of 600 mg, every 12 h, for 14 days, for adults weighing >40 kg. In adolescents and children weighing at least 13 kg, the daily dose is adjusted according to weight. The most common adverse effects are headache, nausea, and gastrointestinal symptoms. There is no need to adjust the dose for patients with liver disease; the drug is not recommended for patients with kidney disease with creatinine clearance <30 mL/min.^6^, ^7^

The efficacy of tecovirimat against Mpox was established in animal models, and there is limited safety and pharmacokinetic data in humans. In these animal-based studies, tecovirimat showed the ability to significantly reduce mortality rates among animals exposed to Mpox, achieving survival rates of no less than 90%.^7^

Mpox causes significant morbidity and mortality, mainly related to unaesthetic scars. Surgical excision, with or without adjuvant treatments, constitutes an efficient and low-cost therapeutic alternative.^3^, ^8^ In the present case, the scars were excised, with satisfactory results (Fig. 4C).

The increase in the number of cases of Mpox has raised concern among health authorities worldwide. Cutaneous manifestations are evident and the role of dermatologists in the early diagnosis is essential. Isolation measures, contact control, mandatory notification, educational and prevention efforts, together with public health policies are essential to reduce the risk of spreading the virus.

Mpox represents a global challenge, requiring greater public awareness of the disease and more studies related to specific treatments and vaccines.

## Financial support

None declared.

## Authors' contributions

Kananda Kesye Sousa Nunes: Design and planning of the study; drafting and editing of the manuscript; critical review of the literature; critical review of the manuscript; approval of the final version of the manuscript.

Carlos Alberto Chirano Rodrigues: Effective participation in research orientation; intellectual participation in the propaedeutic and/or therapeutic conduct of the studied case; critical review of the manuscript; approval of the final version of the manuscript.

Beatriz Costa Cardoso: Drafting and editing of the manuscript; critical review of the literature; critical review of the manuscript; approval of the final version of the manuscript.

Iêda Lúcia Santos Magno: Drafting and editing of the manuscript; critical review of the literature; critical review of the manuscript; approval of the final version of the manuscript.

Mathias Gama de Aguiar Ferreira: Drafting and editing of the manuscript; critical review of the literature; critical review of the manuscript; approval of the final version of the manuscript.

Sinésio Talhari: Design and planning of the study; drafting and editing of the manuscript; effective participation in research orientation; intellectual participation in the propaedeutic and/or therapeutic conduct of the studied case; critical review of the literature; critical review of the manuscript; approval of the final version of the manuscript.

## Conflicts of interest

None declared.
